# CDCA3 Is a Novel Prognostic Biomarker Associated with Immune Infiltration in Hepatocellular Carcinoma

**DOI:** 10.1155/2021/6622437

**Published:** 2021-01-29

**Authors:** Zhihuai Wang, Shuai Chen, Gaochao Wang, Sun Li, Xihu Qin

**Affiliations:** ^1^Graduate School of Dalian Medical University, Dalian Medical University, Dalian 116044, China; ^2^Department of General Surgery, The Affiliated Changzhou No. 2 People's Hospital of Nanjing Medical University, Changzhou 213000, China

## Abstract

Cell division cycle-associated protein-3 (CDCA3) contributes to the regulation of the cell cycle. CDCA3 plays an important role in the carcinogenesis of various cancers; however, the association between CDCA3 expression, prognosis of patients, and immune infiltration in the tumor microenvironment is still unknown. Here, we demonstrated that CDCA3 was differentially expressed between the tumor tissues and corresponding normal tissues using in silico analysis in the ONCOMINE and Tumor Immune Estimation Resource (TIMER) databases. We analyzed the relationship between the expression of CDCA3 and prognosis of patients with hepatocellular carcinoma (HCC) using the Kaplan–Meier plotter database and Gene Expression Profiling Interactive Analysis (GEPIA). Furthermore, we determined the prognostic value of CDCA3 expression using univariate and multivariate analyses. We observed that CDCA3 expression closely correlated with immune infiltration and gene markers of infiltrating immune cells in the TIMER database. CDCA3 was highly expressed in the tumor tissues than in the adjacent normal tissues in various cancers, including HCC. Increased expression of CDCA3 was accompanied by poorer overall survival (OS), relapse-free survival (RFS), progression-free survival (PFS), and disease-specific survival (DSS). The correlation between CDCA3 expression and OS and disease-free survival (DFS) was also studied using GEPIA. CDCA3 expression was associated with the levels of immune cell infiltration and was positively correlated with tumor purity. Moreover, CDCA3 expression was associated with gene markers such as *PD-1*, *CTLA4*, *LAG3*, and *TIM-3* from exhausted T cells, *CD3D*, *CD3E*, and *CD2* from T cells, and *TGFB1* and *CCR8* located on the surface of Tregs. Thus, we demonstrated that CDCA3 may be a potential target and biomarker for the management and diagnosis of HCC.

## 1. Introduction

Hepatocellular carcinoma (HCC) is associated with the second most malignant cancer-related deaths worldwide [1]. Furthermore, it is difficult to obtain a clear diagnosis of HCC during the early stages. Moreover, the existing treatment modalities are not effective in most patients, and its high risk of recurrence often leads to poor prognosis [2]. Therefore, it is necessary to identify effective biomarkers and targets for treating patients with HCC, especially patients undergoing immunotherapy for HCC treatment.

Cell division cycle-associated protein-3 (CDCA3) is frequently upregulated in the tumor tissues and is associated with oncogenic properties in several cancers, such as colorectal [3], prostate [4], non-small-cell lung [5], and gastric cancers [6]; however, its role in the pathogenesis of HCC is unknown. CDCA3 controls translation to influence the cell cycle in the G1 phase as cells cannot transfer from the G2 to M phase without CDCA3 expression [7, 8]. Furthermore, CDCA3 expression possibly influences the proliferation of tumor cells. Currently, some researchers have demonstrated that the E2F transcription factor (E2F) family is the potential biomarkers of human lung carcinoma and human breast cancer [9, 10] and that the runt-related transcription factors (RUNXs) can function as the potential prognostic biomarkers of leukemia in humans [11]. All these studies indicated that the transcription factors which controlled the cell cycle played an important role in the development and progression of human cancers. We supposed that CDCA3 may affect the carcinogenic process of human cancer by influencing the cell cycle. At the same time, researchers are inclined to explore the effect of immune infiltration and tumor microenvironment in tumor progression [12, 13]. Recent studies have indicated that the adaptive immune response plays an important role in the progression of cancer growth. In particular, evaluation of the mutations in immune cells is capable of predicting the outcome of patients with cancerous tumors [14]. Tumor-infiltrating lymphocytes (TILs) contribute to the protective immunity against tumor growth [15], and they are possible independent prognostic biomarkers in some tumors [16]. In fact, the degree of TILs is associated with the prognosis of patients with diverse cancers, including breast [17], colorectal [18], and renal cell cancers [19]. Interestingly, research has indicated that the tumor microenvironment is involved, besides the biological characteristics, in breast cancer [20].

There is an urgent need to identify the immune-related mechanism of carcinogenesis in HCC. Therefore, herein, we focused on determining the effects of CDCA3 expression on immune infiltration that contributes to the tumor microenvironment. Furthermore, we aimed to analyze the expression of CDCA3 and the correlation between CDCA3 expression and prognosis of patients with HCC using bioinformatics analyses. We also explored the relationship between CDCA3 expression and tumor-infiltrating immune cells using the Tumor Immune Estimation Resource (TIMER) database. Finally, we explored the levels of various infiltrating immune cells and the expression of gene markers possessed by tumor-infiltrating immune cells using the TIMER database. Our result showed that CDCA3 expression played an important role in the development of HCC, and its expression was related to the prognosis of HCC. We discovered that the related immune cell infiltration signatures were differently expressed between low and high CDCA3 expression groups. We also found that the higher proportion of CD8+ T cells, CD4+ T cells, and B cells appeared in the high CDCA3 expression group. Our data suggested that the high CDCA3 expression promoted the infiltration of T cells and exhausted these cells, and the patients with high CDCA3 expression might have poorer outcomes by analyzing the information of HCC patients obtained from The Cancer Genome Atlas (TCGA) database. CDCA3 expression might influence the tumor immune infiltration microenvironment by regulating the levels of immune infiltration cells. Collectively, these findings indicated that CDCA3 expression modulated the immune escape and immunosuppressive effects and regulated the tumor-infiltrating immune cells in the tumor progression, thereby demonstrating its potential as a prognostic biomarker for HCC.

## 2. Materials and Methods

### 2.1. ONCOMINE Database

The ONCOMINE database (https://http://www.oncomine.org/resource/login.html) [21] is a platform used for online analysis of tumor data. We utilized this database to analyze the differential expression of CDCA3 in tumor samples of 44 types of human cancers and compared its expression in their corresponding normal samples. The counts of analyzed tumor samples and normal samples of each human cancer were obtained from the ONCOMINE database and are shown in Table S1 (the dataset with giant differences in sample size was deleted). The threshold parameters were as follows: *p* = 0.0001, fold change = 2, and gene rank of the top 10% genes. The *p* value was calculated using Student's *t*-test.

### 2.2. Gene Expression Profiling Interactive Analysis (GEPIA)

GEPIA (http://gepia.cancer-pku.cn/index.html) is an online tool where the RNA-Seq data of 9736 tumor samples and 8587 normal samples in 33 types of cancers were analyzed [22]. It can analyze the expression of target genes, conducts survival analysis in different human cancers, and analyzes the correlation between the targeted gene and other genes in GEPIA. Gene expression profiles in GEPIA are acquired from Genotype-Tissue Expression and TCGA databases [22]. The relationship between CDCA3 expression and overall survival (OS) and disease-free survival (DFS) was estimated by classifying the patients into high and low CDCA3 expression groups based on the median CDCA3 expression value; i.e., high 50% values were classified into the high CDCA3 expression group, whereas low 50% values were classified into the low CDCA3 expression group. And the higher the value of OS and DFS, the better the prognosis of the patient. The *p* value was calculated using the logrank test. Logrank *p* < 0.05 was statistically significant, and the hazards ratio (HR) and 95% confidence interval (CI) were also calculated. Significance of HR referred to the ratio of risk rate produced by high CDCA3 expression to the risk rate produced by low CDCA3 expression, on the premise that *p* < 0.05. The higher the HR value, the bigger the ratio of risk rate produced by high CDCA3 expression on survival. We further verified the relationship between the gene markers of T cells (general) and exhausted T cells and expression of CDCA3 using Spearman's correlation. The results are based on the expression datasets, including TCGA tumor and TCGA normal. *p* < 0.05 was considered significant. Here, the value of *R* was used to represent the correlation coefficient between the gene markers of various infiltrating immune cells and expression of CDCA3 in Table 1.

### 2.3. TIMER Database Analysis

**Figure 1:**
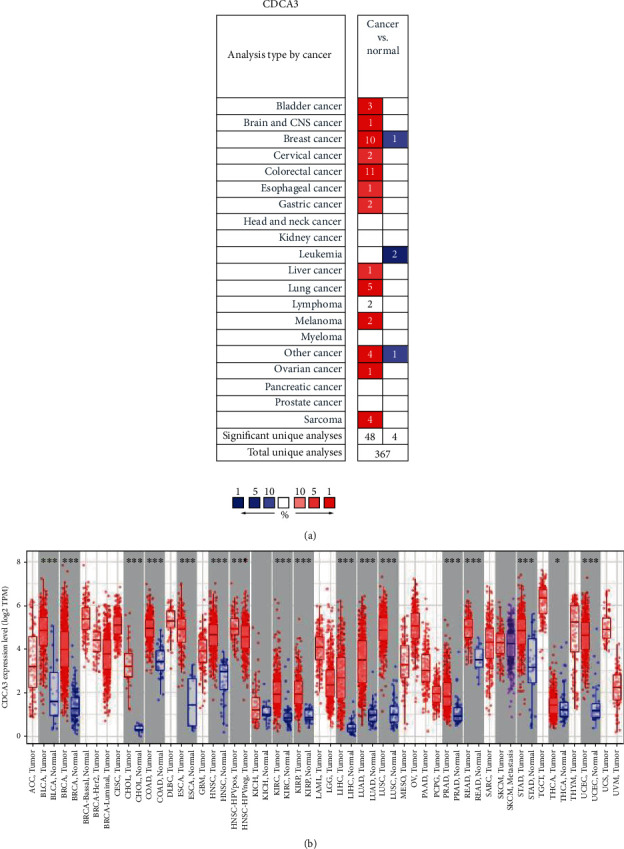
CDCA3 expression in different types of human cancers. (a) High or low expression of CDCA3 in tumor tissues of different human cancers compared with normal tissues using the ONCOMINE database (*p* value of 0.0001, fold change of 2, and gene rank of top 10%.) Note: the number in the blank of (a) represents the amount of the corresponding datasets; the color of the blank in (a) is determined by the best gene rank percentile for the analyses within the cell. (b) The levels of CDCA3 expression in 17 types of human cancers including tumor tissues and normal tissues from TCGA database in TIMER. Note: ^∗^*p* < 0.05, ^∗∗^*p* < 0.01, and ^∗∗∗^*p* < 0.001.

The web server TIMER (https://cistrome.shinyapps.io/timer/) is a comprehensive resource for conducting the systematic analysis of immune infiltrates across 32 cancer types [23]. The abundance of six immune cell infiltrates (B cells, CD4+ T cells, CD8+ T cells, neutrophils, macrophages, and dendritic cells) is estimated with more than 1000 samples using the TIMER algorithm, which has been reported in TCGA [24]. Furthermore, the association between the expressed genes, tumor-infiltrating immune cells, and gene markers of immune infiltrating cells can also be investigated using TIMER [25]. We detected the differential expression of CDCA3 between the tumor and normal tissues in 17 types of human cancers using the “diff Exp” module (Figure 1(a) and Table 2). We analyzed the relationship between CDCA3 expression and abundance of immune infiltrates using the “gene” module. The association between CDCA3 expression and gene markers of infiltrating immune cells was analyzed using the “correlation” module. The infiltrating immune cells included T cells, B cells, TAMs, monocytes, M1 macrophages, M2 macrophages, natural killer (NK) cells, neutrophils, dendritic cells (DCs), T-helper (Th) cells, T-helper 17 (Th17) cells, follicular helper T (Tfh) cells, exhausted T cells, and Tregs. Here, we established a standard to describe the association between CDCA3 expression and gene markers of infiltrating immune cells, where 0.00–0.29 was considered weak, 0.30–0.59 was considered moderate, 0.60–0.79 was considered strong, and 0.80–1.00 was considered very strong expression [26]. The correlation was analyzed by Spearman's correlation analysis. CDCA3 expression was visualized with log2 TPM in the scatter plots, the *x*-axis represented the CDCA3 expression, and the *y*-axis represented the infiltrating levels. The value of partial correlation coefficient (partial cor) reflected the degree of correlation between the expression of CDCA3 and immune infiltration. *p* < 0.05 was statistically significant.

### 2.4. Kaplan–Meier Plotter Database

**Figure 2:**
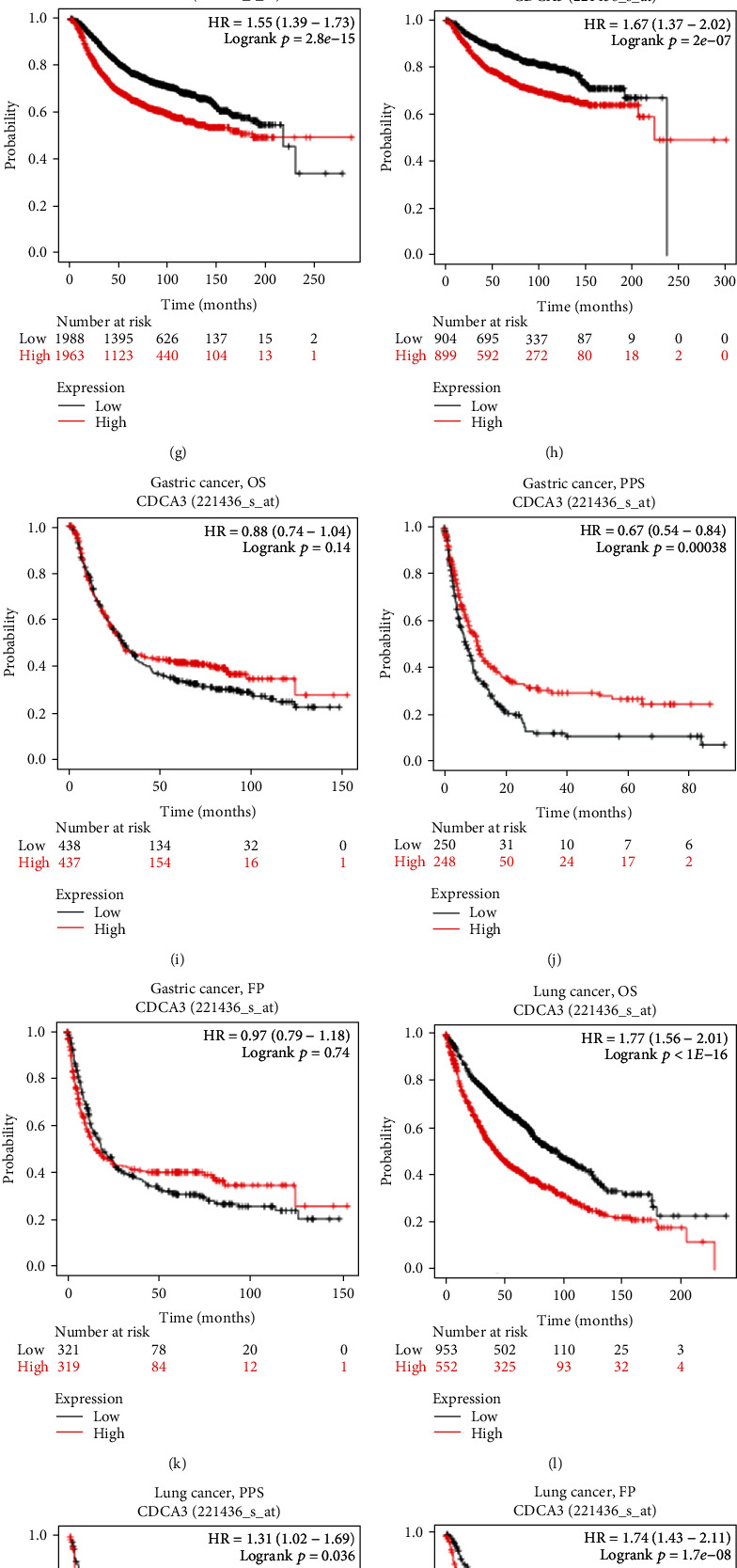
Kaplan–Meier survival curve analysis of the prognostic significance of high and low expression of CDCA3 in different types of human cancers using the Kaplan–Meier plotter database. (a–d) Survival curves of OS, PFS, RFS, and DSS (*n* = 364, *n* = 370, *n* = 316, and *n* = 362) in HCC. (e–h) Survival curves of OS, PPS, RFS, and DMFS (*n* = 1402, *n* = 414, *n* = 3951, and *n* = 1803) in the breast cancer. (i–k) Survival curves of OS, PPS, and FP (*n* = 875, *n* = 498, and *n* = 640) in gastric cancer. (l–n) Survival curves of OS, PPS, and FP (*n* = 1925, *n* = 344, and *n* = 982) in lung cancer. (o–q) Survival curves of OS, PFS, and PPS (*n* = 1656, *n* = 1135, and *n* = 782) in ovarian cancer. OS: overall survival; PFS: progression-free survival; RFS: relapse-free survival; DSS: disease-specific survival; DMFS: distant metastasis-free survival; PPS: postprogression survival; FP: first progression. Note: HR (hazard ratio) represents the ratio of the risk rate produced by the application of high expression of CDCA3 to the risk rate produced by low expression of CDCA3 on survival; logrank *p* < 0.05 was statistically significant; the counts of patients at risk were listed under the survival plots.

The correlation between the expression of more than 54,000 genes and patient survivals who suffered from 21 types of different cancers was analyzed based on 10,000 cancer samples in the Kaplan–Meier plotter database (http://kmplot.com/analysis) [27], and the relationship between CDCA3 expression and prognosis of HCC patients was analyzed by the database as well, in which 371 liver samples, 1440 gastric samples, 3452 lung samples, 2190 ovarian samples, and 6234 breast cancer samples were contained. The median survival, including overall survival (OS), progression-free survival (PFS), relapse-free survival (RFS), disease-specific survival (DSS), distant metastasis-free survival (DMFS), postprogression survival (PPS), and first progression (FP), in different cancers was computed using the Kaplan–Meier survival plot by categorizing the patients (the counts of patients are listed under the survival plots in Figure 2) into two cohorts according to the median CDCA3 expression value, and the higher the value, the better the prognosis of the patient. We chose the best probe set of CDCA3 to compute the Kaplan–Meier survival plots, and the prognostic value of CDCA3 in HCC patients with diverse clinicopathologic features was also analyzed by the Kaplan–Meier plotter database; the clinicopathologic features are listed in Table 2. The *p* value was calculated by using the logrank test. Logrank *p* value, hazards ratio (HR) with 95% CI, and survival plots were all obtained from the database. Here, logrank *p* < 0.05 was statistically significant, and significance of HR referred to the ratio of risk rate produced by the application of high expression of CDCA3 to the risk rate produced by low expression of CDCA3. Prognosis of the patients was affected by the HR value (the higher the HR, the poorer the prognosis).

### 2.5. Univariate and Multivariate Analyses of CDCA3 Expression on Survival

We employed the univariate and multivariate analyses to analyze the independent predictive value of CDCA3 expression and the other clinicopathologic features on survival. Transcriptome profiling data of primary HCC (LIHC) and clinical information of related patients were downloaded from TCGA website (https://portal.gdc.cancer.gov/repository). The data comprised the transcriptome profile of 50 normal tissue samples and 374 tumor tissue samples. The workflow type was HTSeq-FPKM, and the clinicopathologic features, including age, gender, grade, stage, T classification, M classification, and N classification, were all sorted by the Perl programming language. The CDCA3 expression information was extracted using R (version 4.0.3) packages “biocmanager” and “limma.” Then, we matched the clinical data with the CDCA3 expression data, and we estimated the unknown or incomplete data of clinical data. As a consequence, the Cox proportional hazard regression model was employed. Univariate analysis was conducted using the R “survival” package, and multivariate analysis was carried out using R “survminer” and “survival” packages. They were both analyzed with the coxph and ggforest commands. The hazard ratio and 95% confidence interval (CI) were both computed through the procedure. Here, the hazard ratio (HR) referred to the ratio of risk rate of survival produced by applying a certain clinicopathologic feature to the risk rate of survival produced without applying that clinicopathologic feature. The higher the values of HR, the prognosis of the patient was affected more by that clinicopathologic feature. *p* < 0.05 was statistically significant for the Cox regression analyses. Median CDCA3 expression was set as the threshold value.

### 2.6. Statistical Analysis

The differential expression of CDCA3 between tumor tissue and normal tissue was calculated by Student's *t*-test, and the results were ultimately shown by rank (%), *p*, value and fold changes. Relationship between CDCA3 expression and clinicopathologic features, including grade and stage of tumor tissues, was analyzed with R (version 4.0.3) using the Kruskal–Wallis test, where *p* < 0.05 was considered statistically significant. Prognostic curves were plotted in the Kaplan–Meier plotter and GEPIA. The logrank test and HR analyses were used to examine the statistical difference between survival curves in HCC with low and high expression of CDCA3, and the median of CDCA3 expression was the threshold for splitting highly expressed CDCA3 and lowly expressed CDCA3. The data are presented as mean ± standard error of mean. The association between the CDCA3 expression, the abundance of immune infiltrates, and the gene markers of infiltrating immune cells was analyzed by Spearmen' correlation analysis and statistical significance in TIMER and GEPIA databases, and *p* < 0.05 was considered statistically significant.

## 3. Results

### 3.1. Expression of CDCA3 in Various Human Cancers

According to the results from the analysis using ONCOMINE, we observed that CDCA3 mRNA expression was significantly elevated in cancer tissue samples from bladder cancer, brain and central nervous system cancer, medullary breast carcinoma, ductal breast carcinoma, ductal breast carcinoma in situ epithelia, lobular breast carcinoma, cervical cancer, colorectal cancer, esophageal cancer, gastric cancer, liver cancer, lung cancer, lymphoma, melanoma, sarcoma, malignant fibrous histiocytoma, skin squamous cell carcinoma, and vulvar intraepithelial neoplasia in comparison with their corresponding normal tissue samples; however, CDCA3 mRNA expression was lower in invasive breast carcinoma stroma, acute myeloid leukemia, and teratoma-NOS (the nonspecific type of teratoma) samples than in normal tissue sample (Figure 1(a) and Table S1). We verified our results using TIMER, and our results were validated as CDCA3 expression was different between tumor and normal tissue samples. Expression of CDCA3 was higher in bladder urothelial carcinoma (BLCA), breast invasive carcinoma (BRCA), cholangiocarcinoma (CHOL), colon adenocarcinoma (COAD), esophageal carcinoma (ESCA), head and neck squamous cell carcinoma (HNSC), kidney renal clear cell carcinoma (KIRC), kidney renal papillary cell carcinoma (KIRP), liver hepatocellular carcinoma (LIHC), lung adenocarcinoma (LUAD), lung squamous cell carcinoma (LUSC), and prostate adenocarcinoma (PRAD) than in their corresponding normal tissues (Figure 1(b)). The two databases both confirmed that CDCA3 was highly expressed in HCC.

### 3.2. Prognostic Analysis of CDCA3 in Various Human Cancers

We used the Kaplan–Meier plotter database to evaluate the interaction between CDCA3 expression and prognosis in various human cancers. Higher values of OS, PFS, RFS, DSS, DMFS, PPS, and FP indicated better survival in patients with HCC. The higher the value of HR, the greater the prognosis of the patient, on the premise that *p* < 0.05. We observed that higher CDCA3 expression was associated with poorer OS, PFS, DSS, and RFS in HCC (OS: HR = 1.72, 95%CI = 1.21–2.45, *p* = 0.002; PFS: HR = 1.55, 95%CI = 1.15–2.08, *p* = 0.0034; DSS: HR = 2.13, 95%CI = 1.35–3.36, *p* = 0.00086; and RFS: HR = 1.55, 95%CI = 1.12–2.16, *p* = 0.0086; Figures 2(a)–2(d)), poorer OS, RFS, and DMFS in breast cancer (OS: HR = 1.78, 95%CI = 1.43–2.22, *p* = 1.4*E* − 07; RFS: HR = 1.55, 95%CI = 1.39–1.73, *p* = 2.8*E* − 15; and DMFS: HR = 1.67, 95%CI = 1.37–2.02, *p* = 2*E* − 07; Figures 2(e)–2(h)), better PPS in gastric cancer (PPS: HR = 0.67, 95%CI = 0.54–0.84, *p* = 0.00038; Figure 2(j)), and poorer OS, PPS, and FP in lung cancer (OS: HR = 1.77, 95%CI = 1.56–2.01, *p* < 1*E* − 16; PPS: HR = 1.31, 95%CI = 1.02–1.69, *p* = 0.036; and FP: HR = 1.74, 95%CI = 1.43–2.11, *p* = 1.7*E* − 08; Figures 2(l)–2(n)); however, CDCA3 expression had no correlation with PPS (HR = 1.24, 95%CI = 0.97–1.58, *p* = 0.087) in breast cancer (Figure 2(f)); OS (HR = 0.88, 95%CI = 0.74–1.04, *p* = 0.14) and FP (HR = 0.97, 95%CI = 0.79–1.18, *p* = 0.74) in gastric cancer (Figures 2(i)–2(k)); and OS (HR = 1.13, 95%CI = 1–1.29, *p* = 0.058), PFS (HR = 1.02, 95%CI = 0.9–1.15, *p* = 0.8), and PPS (HR = 1.13, 95%CI = 0.95–1.33, *p* = 0.17) in ovarian cancer (Figures 2(o)–2(q)). According to the results, we found that the high expression of CDCA3 was correlated with the poor prognosis in HCC, breast cancer, and lung cancer (HR > 1, *p* < 0.05). As revealed by PPS (HR = 0.67, 95%CI = 0.54–0.84, *p* = 0.00038) (Figure 2(j)), the high CDCA3 expression was associated with the better prognosis in gastric cancer because the HR < 1 and *p* < 0.05. We also used GEPIA to assess the relationship between CDCA3 expression and prognosis of the patients in 33 different TCGA cancer types (Figure S1). High expression of CDCA3 was correlated with poorer OS and DFS in adrenocortical carcinoma (ACC), KIRC, KIRP, brain lower grade glioma (LGG), LIHC, LUAD, mesothelioma (MESO), and sarcoma (SARC); poorer DFS in kidney chromophobe (KICH), pheochromocytoma and paraganglioma (PCGP), PRAD, uterine corpus endometrial carcinoma (UCEC), and uveal melanoma (UVM); and poorer OS in pancreatic adenocarcinoma (PAAD) and skin cutaneous melanoma (SKCM). These results indicated that a high expression of CDCA3 had a strong association with poor outcomes for patients with various cancers, especially in HCC, and the correlation depended on the type of tumor.

### 3.3. Association between CDCA3 Expression and Clinicopathologic Features of HCC

**Figure 3:**
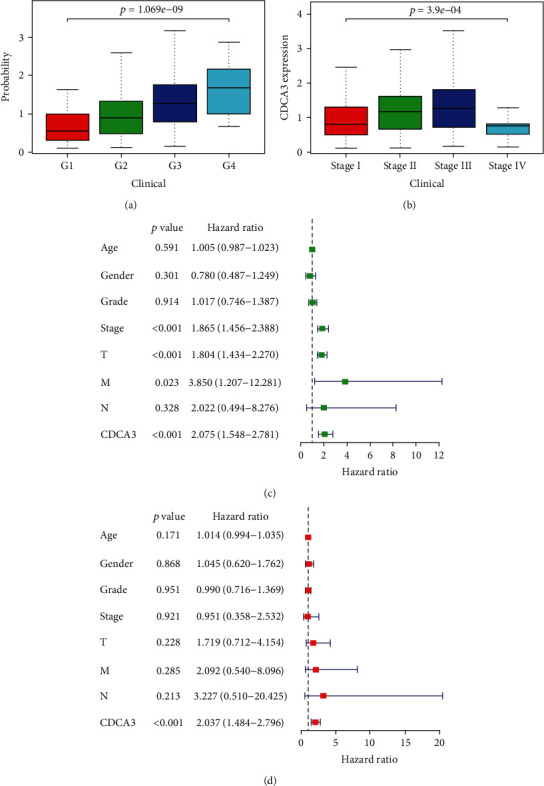
(a) The correlation between the CDCA3 expression and the grade in HCC. (b) The correlation between the CDCA3 expression and the stage in HCC. (c) Univariate analysis for the prognostic value of the CDCA3 expression and clinicopathologic features in HCC. (d) Multivariate analysis for the independent prognostic value of the CDCA3 expression and clinicopathologic features in HCC. Note: the hazard ratio represents the ratio of the risk rate produced by the application of a certain clinicopathologic feature to the risk rate produced without the clinicopathologic feature on survival; *p* < 0.05 was significantly different. T: T classification; N: N classification; M: M classification.

In this section, we aimed to figure out the association between the expression of CDCA3 and clinicopathologic features. The RNA sequence data and corresponding clinicopathologic features from 50 normal tissue samples and 374 tumor tissue samples were downloaded from TCGA database. The results showed that expression of CDCA3 was significantly correlated with the tumor grade (*p* = 1.069*E* − 09; Figure 3(a)) and stage (*p* = 3.9*E* − 04; Figure 3(b)). With the change of the grade and the stage, the expression level of CDCA3 showed a corresponding tendency of changing. To further analyze the impact of CDCA3 expression on patient prognosis with different clinicopathologic features (the clinicopathologic features included the stage, the grade, the AJCC_T, the vascular invasion, the gender, the race, the sorafenib treatment, and the alcohol consumption and hepatitis virus), we used the Kaplan–Meier plotter database and observed that higher expression of CDCA3 was associated with poorer OS and PFS in both females (OS: HR = 2.17, *p* = 0.0085; PFS: HR = 2.19, *p* = 0.0052) and males (OS: HR = 1.81, *p* = 0.0095; PFS: HR = 1.76, *p* = 0.0024), Whites (OS: HR = 2.48, *p* = 0.0059; PFS: HR = 2.02, *p* = 0.0016), Asians (OS: HR = 3.01, *p* = 0.00048; PFS: HR = 1.91, *p* = 0.007), and patients without viral hepatitis infections (OS: HR = 2.18, *p* = 0.00073; PFS: HR = 2.67, *p* = 0.000011) (Table 2). Interestingly, high CDCA3 expression levels were associated with poor OS and PFS in stages I+II, II+III, III, and III+IV; AJCC-T II and III stages; and grades I and II in HCC (Table 2). *p* < 0.05 was statistically significant, and the hazard ratio > 1 represented the higher risk factors produced by high CDCA3 expression affected in the prognosis of patients with different clinicopathologic features. These results indicated that CDCA3 expression was in high correlation with the outcome of patients with HCC through females, males, Whites, Asians, patients without viral hepatitis infections, stages (I+II, II+III, III, and III+IV), AJCC-T stages (II and III), and grades (I and II).

### 3.4. Univariate and Multivariate Analyses of CDCA3 Expression on Survival

We analyzed the relationship between patient survival and CDCA3 expression using the univariate and multivariate analyses. Univariate analysis showed that T stage (HR = 1.804; 95%CI = 1.434–2.270; *p* < 0.001), M stage (HR = 3.850; 95%CI = 1.207–12.281; *p* = 0.023), and CDCA3 expression (HR = 2.075; 95%CI = 1.548–2.781; *p* < 0.001) effectively predicted the survival of HCC patients (Figure 3(c)). It was remarkable that the hazard ratio (HR) of CDCA3 expression in univariate analysis equaled to 2.075, the value of HR and the *p* < 0.001 both indicated that CDCA3 can predict the prognosis in HCC, and the hazard ratio revealed that the patients with high CDCA3 expression had 2.075 times of higher risk in poor OS than the patients with low CDCA3 expression in univariate analysis. To further verify the prognostic value of CDCA3 expression, we used the multivariate analysis. The results showed that high expression of CDCA3 was associated with poor outcomes in HCC patients, and it could act as a potential independent predictor of survival (HR = 2.037; 95%CI = 1.484–2.796; *p* < 0.001; Figure 3(d)) by excluding confounding factors. Besides, the value of the hazard ratio revealed that the patients with high CDCA3 expression had 2.037 times of higher risk in poor OS than the patients with low CDCA3 expression.

### 3.5. CDCA3 mRNA Expression Interacted with the Levels of Infiltrating Immune Cells in HCC

**Figure 4:**
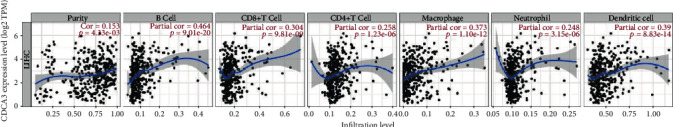
Correlation analysis of CDCA3 expression and infiltration levels of immune cells in HCC tissues using the TIMER database. The expression of CDCA3 was positively correlated with tumor purity and infiltration levels of immune cells in HCC tissues. The abundances of six infiltrating immune cells included B cells, CD4+ T cells, CD8+ T cells, neutrophils, macrophages, and dendritic cells. Note: purity represents the tumor purity; partial cor represents the correlation coefficient between the infiltrating levels of immune cells and the expression of CDCA3; the higher the value of partial cor, the stronger the correlation coefficient; *p* < 0.05 was statistically significant.

TILs can lead to a poor outcome in patients with cancer [28]. We explored the relationship between the infiltration levels of immune cells and CDCA3 expression in 39 types of human cancer using the TIMER database, where the value of “partial cor” reflected the degree of correlation between CDCA3 expression and immune infiltration. The abundances of six infiltrating immune cells included B cells, CD4+ T cells, CD8+ T cells, neutrophils, macrophages, and dendritic cells. The tumor purity of clinical samples can influence the analysis of immune infiltration at the genomic aspect [29]. Among them, the CDCA3 expression was correlated with tumor purity in 22 cancer types, the CDCA3 expression was also associated with the infiltrating levels of B cells in 13 cancer types, CD8+ T cells in 11 cancer types, CD4+ T cells in 15 cancer types, macrophages in 17 cancer types, neutrophils in 12 cancer types, and dendritic cells in 14 cancer types (Figure 4 and Figure S2). We chose the type of cancer whose expression of CDCA3 showed a strong correlation with both the patients' prognosis in GEPIA and tumor purity in TIMER. The results showed that high expression of CDCA3 was associated with poor prognosis of patients, high levels of infiltrating immune cells, and tumor purity in HCC and ACC. CDCA3 expression was positively correlated with the infiltrating levels of B cells (partial cor = 0.464, *p* = 9.01*E* − 20), CD8+ T cells (partial cor = 0.304, *p* = 9.81*E* − 09), CD4+ T cells (partial cor = 0.258, *p* = 1.23*E* − 06), macrophages (partial cor = 0.373, *p* = 1.10*E* − 12), neutrophils (partial cor = 0.248, *p* = 3.15*E* − 06), and dendritic cells (partial cor = 0.39, *p* = 8.83*E* − 14; Figure 4) in HCC. Notably, the expression of CDCA3 was also associated with tumor purity (partial cor = 0.153, *p* = 4.33*E* − 03; Figure 4), and it showed that the CDCA3 expression had a positive correlation with tumor purity in HCC. The high CDCA3 expression was also correlated with a poorer survival (OS: HR = 2.5, *p* = 6.4*E* − 07; DFS: HR = 1.8, *p* = 0.00026) (Figure S1 ac-ad) but was positively related to the infiltrating levels of B cells (partial cor = 0.252, *p* = 2.42*E* − 08), CD4+ T cells (partial cor = 0.222, *p* = 1.01*E* − 06), macrophages (partial cor = 0.207, *p* = 5.93*E* − 06), neutrophils (partial cor = 0.174, *p* = 1.35*E* − 04), and dendritic cells (partial cor = 0.229, *p* = 4.54*E* − 07) in LGG (Figure S2 s). These results suggested that CDCA3 expression regulated the infiltration of immune cells into tumor tissues in HCC and LGG. Therefore, CDCA3 expression was associated with tumor-infiltrating immune cells and caused poor outcomes depending on the type of tumor-infiltrating immune cells in HCC.

### 3.6. Association Analysis between mRNA Levels of CDCA3 and Gene Markers of Various Immune Cells

Immune cells play an essential role in angiogenesis and regulating immune escape in tumor progression. Tregs have an impact on immune escape, angiogenesis, and immune checkpoint regulation, which is essential for the cancer-killing effect mediated by T cells [28]. Therefore, we explored the correlation between the expression of CDCA3 and gene markers of different immune cells, such as B cells, neutrophils, monocytes, tumor-associated macrophages (TAMs), M1 and M2 macrophages, CD4+ T cells, CD8+ T cells, natural killer cells (NK cells), dendritic cells, and different T cells, including Tregs, T-helper 1 cells (Th1), T-helper 2 cells (Th2), T-helper 17 cells (Th17), follicular helper T cells (Tfh), and exhausted T cells.

**Figure 5:**
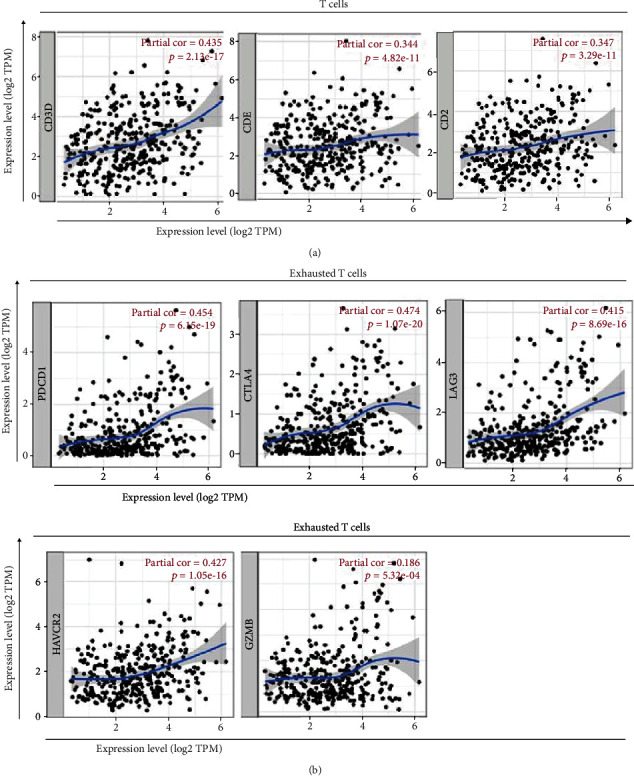
Correlation analysis of CDCA3 expression and the gene markers of infiltrating immune cells in HCC using the TIMER database. (a) The scatter plots show the correlation between CDCA3 expression and the gene markers of T cells (CD3D, CD3E, and CD2). (b) The scatter plots show the correlation between CDCA3 expression and the gene markers of exhausted T cells (PDCD1, CTLA4, LAG3, HAVCR2, and GZMB). Note: partial cor both represents the correlation coefficient between the gene markers of T cells and the expression of CDCA3 as well as the correlation coefficient between exhausted T cells and the expression of CDCA3; the higher the value of partial cor, the stronger the correlation coefficient; *p* < 0.05 was statistically significant.

Our results showed that the expression of CDCA3 was significantly associated with gene markers of all subsets of T cells (CD3D: partial cor = 0.435, *p* = 2.13*E* − 17; CD3E: partial cor = 0.344, *p* = 4.82*E* − 1; and CD2: partial cor = 0.347, *p* < 0.0001) adjusted by tumor purity (Figure 5(a) and Table 3), which indicated that CDCA3 was related to cytokine secretion. CDCA3 expression was associated with gene markers of exhausted T cells, such as *PD-1* (partial cor = 0.454, *p* = 6.18*E* − 19), *CTLA4* (partial cor = 0.474, *p* = 1.07*E* − 20), *LAG3* (partial cor = 0.415, *p* = 8.69*E* − 16), and *TIM-3* (partial cor = 0.427, *p* = 1.05*E* − 16; Figure 5(b) and Table 3). Furthermore, CDCA3 expression was also related to *CCR8* (partial cor = 0.352, *p* < 0.0001) and *TGFB1* (partial cor = 0.351, *p* < 0.0001) on the surface of Tregs (Table 3). The values of partial cor reflect the degree of the correlation coefficient between the expression of CDCA3 and the expression of gene markers that belongs to the infiltrating immune cells, and *p* < 0.05 was statistically significant. Some studies have mentioned gene markers for tumor-infiltrating immune cells [30]. Thus, CDCA3 can lead to immunosuppression and is associated with poor prognosis in HCC. We obtained similar results pertaining to correlations between the expression of CDCA3 and gene markers of infiltrating immune cells using GEPIA (Table 1). The results showed that the CDCA3 expression was correlated with the gene markers of T cells (general), of which CD3D (tumor, *R* = 0.33, *p* = 1.4*E* − 10; normal, *R* = 0.34, *p* = 0.016), CD3E (tumor, *R* = 0.19, *p* = 0.00034; normal, *R* = 0.32, *p* = 0.025), and CD2 (tumor, *R* = 0.21, *p* = 4.7*E* − 05; normal, *R* = 0.31, *p* = 0.028) in both the tumor tissue dataset and normal tissue dataset from TCGA were chosen (Table 1). The expression of CDCA3 was also associated with the gene markers of exhausted T cells, of which PD-1 (tumor, *R* = 0.35, *p* = 3.4*E* − 12), CTLA4 (tumor, *R* = 0.35, *p* = 3.1*E* − 12), LAG3 (tumor, *R* = 0.34, *p* = 3.4*E* − 11), TIM-3 (tumor, *R* = 0.27, *p* = 1.1*E* − 07), and GZMB (tumor, *R* = 0.1, *p* = 0.048) in the tumor tissue dataset from TCGA were chosen (Table 1). The value of *R* represented the degree of the correlation coefficient between the expression of CDCA3 and gene markers of infiltrating immune cells, on the premise that *p* < 0.05. We found that the gene markers of the T cells (general) and the exhausted T cells were strongly correlated with CDCA3 expression due to a higher value of *R*. All these revealed that CDCA3 was highly correlated with T cells, exhausted T cells, and Treg, but the expression of CDCA3 was not associated with the gene markers of Tfh and M2 macrophage (Table 3). Taken together, CDCA3 expression may have participated in the regulation of immune checkpoint and immune escape, and its expression closely correlated with poor outcomes in patients with HCC.

## 4. Discussion

CDCA3 is widely known as a trigger of mitotic entry. It is expressed in many tumor tissues and plays an essential role in tumorigenesis and tumor growth in various cancers. Recent studies have shown that CDCA3 functions as a crucial oncogene and is associated with poor outcomes in patients with gastric cancer, and it is a potential therapeutic target in the management of gastric cancer [6]. CDCA3 can regulate E2F1 expression to influence growth and progression [31]. Many studies have elucidated the role of CDCA3 expression in diverse tumors; for instance, CDCA3 inhibits the formation of tumors by affecting cell growth and inducing apoptosis in pancreatic cancer [32]. CDCA3 expression affects other cancers as well, such as renal cell carcinoma [33], breast cancer [34], acute myeloid leukemia [35], and non-small-cell lung cancer [5]. Recently, some studies have suggested that CDCA3 is expressed differently between normal tissues and tumor tissues; however, only few studies have explained the underlying mechanism and pathways of CDCA3 expression in HCC. In this study, we analyzed the expression of CDCA3 and prognosis of patients with HCC using in silico analysis. To the best of our knowledge, this is the first study to discuss the prognostic value of CDCA3 and its correlation with immune infiltration in HCC. We observed higher CDCA3 expression in tumor tissues than in corresponding normal tissues, and higher CDCA3 expression resulted in poorer outcomes in patients with HCC. According to the univariate and multivariate analyses, we identified that T stage, M stage, and CDCA3 expression had significant prognostic values for predicting the survival of patients with HCC; in fact, the high expression determined poor OS of patients with HCC and suggested that increased CDCA3 expression deteriorated the state of patients with HCC. Next, we determined that the expression of CDCA3 was related to the infiltration of immune cells and gene markers of immune infiltrating cells, especially in CD8+ T cells, CD4+ T cells, B cells, T cells, and exhausted T cells. Collectively, these results provide evidence that CDCA3 is involved in immune escape and immunosuppression in the tumor microenvironment.

An immunosuppressive microenvironment can lead to immune escape and immune tolerance by activating different pathways in HCC [36]. We observed that the expression of CDCA3 correlated with immune infiltration and was especially involved with the gene markers of Tregs and exhausted T cells. Thus, CDCA3 expression can potentially influence the immunosuppressive effect in HCC.

Morbidity and lethality because of HCC have increased over the years, and identification of a biomarker for diagnosis and prognosis is necessary. We determined that CDCA3 expression was closely related to gene markers of T cells and exhaustion-related inhibitory receptors. An increase in the inhibitory receptors can restrain the immune response and function of T cells during carcinogenesis in HCC [13–16], and the inhibition of anti-immune checkpoints can lead to a better outcome in patients [37]. Thus, CDCA3 can lead to poor prognosis through the modulation of immune escape and immunosuppression effects and regulation of tumor-infiltrating immune cells. Consequently, CDCA3 may be a potential target and biomarker for the management and diagnosis of HCC, respectively.

However, this study has several limitations. First, bioinformatics methods can only provide a way to explore the underlying mechanism in HCC with limited reliability, and thus, our results require further verification using in vitro and in vivo experiments. Furthermore, the correlation coefficients that described the relationship between CDCA3 expression and gene markers of different immune infiltrating cells mostly showed a weak and moderate correlation [26, 30]. Thus, it is necessary to compare the effectiveness of assessing CDCA3 expression with current traditional biomarkers and explore the functions and underlying mechanism of CDCA3 expression in HCC.

## 5. Conclusion

Compared with the corresponding normal tissues, the expression of CDCA3 was upregulated in tumor tissues of various human cancers including HCC. Prognostic values of CDCA3 expression were significantly observed using the Kaplan–Meier plotter analysis and GEPIA database. High expression of CDCA3 was associated with poor outcome for patients with various cancers, and elevated CDCA3 expression led to poor OS, RFS, PFS, and DSS in HCC. High expression of CDCA3 also correlated with poor OS and DFS in ACC, KIRC, KIRP, LGG, LIHC, LUAD, MESO, and SARC in GEPIA. According to the results of the univariate and multivariate analyses, T stage, M stage, and CDCA3 expression were important prognostic factors for the survival of patients with HCC; importantly, high CDCA3 expression had the potential to be an independent predictor for poor outcome for patients with HCC according to the results of multivariate analyses. Furthermore, CDCA3 expression was strongly associated with the infiltration of immune cells, including B cells, neutrophils, monocytes, TAMs, M1 and M2 macrophages, CD4+ T cells, CD8+ T cells, NK cells, and dendritic cells; CDCA3 expression was positively associated with tumor purity and correlated with gene markers of T cells (general), exhausted T cells, and Tregs. Thus, CDCA3 is an important prognostic biomarker for patients with HCC, and it is closely associated with immune infiltration. However, the underlying mechanisms and pathways related to CDCA3 expression need to be explored further.

## Figures and Tables

**Table 1:** Correlation analysis between CDCA3 and gene markers of immune cells in GEPIA.

Description	Gene markers	HCC				
		Tumor			Normal	
		*R*	*p* value		*R*	*p* value
T cell (general)	CD3D	0.33	1.4**e** − 10		0.34	**0.016**
	CD3E	0.19	**0.00034**		0.32	**0.025**
	CD2	0.21	4.7**e** − 05		0.31	**0.028**
Exhausted T cell	PD-1	0.35	3.4**e** − 12		0.27	0.057
	CTLA4	0.35	3.1**e** − 12		0.27	0.061
	LAG3	0.34	3.4**e** − 11		0.15	0.31
	TIM-3	0.27	1.1**e** − 07		0.57	-0.081
	GZMB	0.1	**0.048**		0.25	0.077

Note: *R* represents the correlation coefficient between the gene markers of various infiltrating immune cells and the expression of CDCA3; tumor represents the tumor tissue dataset (LIHC) from TCGA; normal represents the normal tissue dataset (LIHC) from TCGA; bold values indicate *p* < 0.05; *p* < 0.05 was statistically significant.

**Table 2:** Correlation of CDCA3 mRNA expression and prognosis in hepatocellular carcinoma with different clinicopathologic features by the Kaplan–Meier plotter.

Clinicopathologic features	Overall survival			Progression-free survival		
	*N*	Hazard ratio	*p* value	*N*	Hazard ratio	*p* value
Sex						
Female	118	2.17 (1.24-3.81)	**0.0058**	121	2.19 (1.25-3.84)	**0.0052**
Male	246	1.81 (1.15-2.84)	**0.0095**	249	1.76 (1.21-2.54)	**0.0024**
Stage						
I	170	1.95 (0.97-3.89)	0.055	171	1.64 (1-2.71)	**0.049**
II	83	4.39 (1.5-12.83)	**0.0031**	84	1.76 (0.92-3.37)	0.082
I+II	253	2.33 (1.32-4.11)	**0.0026**	254	1.52 (1.04-2.23)	**0.029**
II+III	166	2.56 (1.51-4.34)	**0.00031**	167	1.95 (1.23-3.1)	**0.0041**
III	83	2.32 (1.27-4.22)	**0.0047**	83	2.05 (1.09-3.86)	**0.022**
III+IV	87	2.18 (1.22-3.9)	**0.0074**	88	2.18 5(1.17-4.08)	**0.012**
V	4	—	—	5	—	—
Grade						
1	55	4.97 (1.73-14.25)	**0.0013**	55	2.8 (1.16-6.79)	**0.019**
2	174	1.97 (1.13-3.44)	**0.015**	175	2.15 (1.39-3.34)	**0.00044**
3	118	2.22 (1.21-4.09)	**0.0085**	119	1.67 (0.99-2.83)	0.051
4	12	—	—	12	—	—
AJCC_T						
1	180	1.87 (0.98-3.56)	0.055	180	1.63 (1-2.65)	**0.047**
2	90	3.69 (1.4-9.7)	**0.0047**	92	1.99 (1.07-3.69)	**0.026**
3	78	2.31 (1.25-4.25)	**0.0059**	78	2.25 (1.08-4.68)	**0.026**
4	13	—	—	13	—	—
Vascular invasion						
None	203	2.09 (1.14-3.81)	**0.015**	204	1.47 (0.94-2.3)	0.089
Micro	90	2.15 (0.86-5.35)	0.092	91	1.57 (0.85-2.9)	0.14
Macro	16	—	—	16	—	—
Race						
White	181	2.48 (1.27-4.84)	**0.0059**	183	2.02 (1.29-3.14)	**0.0016**
Black or African man	17	—	—	17	—	—
Asian	155	3.01 (1.57-5.76)	**0.00048**	155	1.91 (1.18-3.08)	**0.007**
Alcohol consumption						
Yes	115	1.41 (0.75-2.67)	0.29	115	1.97 (1.17-3.34)	**0.0099**
None	202	1.63 (1.03-2.59)	**0.037**	204	1.47 (0.98-2.19)	0.06
Virus hepatitis						
Yes	150	1.19 (0.62-2.27)	0.6	152	0.96 (0.61-1.52)	0.86
None	167	2.18 (1.37-3.45)	**0.00073**	167	2.67 (1.69-4.21)	1.1**e** − 05

Note: *N* represents the numbers of patients with different clinicopathologic features; the hazard ratio represents the higher risk factors produced by high CDCA3 expression affected in the prognosis of patients with different clinicopathologic features; clinicopathologic features include stage, grade, AJCC_T, vascular invasion, gender, race, sorafenib treatment, alcohol consumption, and hepatitis virus; bold values indicate *p* < 0.05; *p* < 0.05 was statistically significant.

**Table 3:** Correlation analysis between CDCA3 and related genes and markers of immune cells in TIMER.

Description	Gene markers	HCC			
		None		Purity	
		Partial cor	*p* value	Partial cor	*p* value
CD8+ T cell	CD8A	0.189	∗∗∗	0.294	∗∗∗
	CD8B	0.225	∗∗∗	0.326	∗∗∗
T cell (general)	CD3D	0.318	∗∗∗	0.435	∗∗∗
	CD3E	0.194	∗∗	0.344	∗∗∗
	CD2	0.209	∗∗∗	0.347	∗∗∗
B cell	CD19	0.243	∗∗∗	0.319	∗∗∗
	CD79A	0.154	∗	0.266	∗∗∗
Monocyte	CD86	0.259	∗∗∗	0.395	∗∗∗
	CD115	0.109	0.0366	0.228	∗∗∗
TAM	CCL2	-0.001	0.979	0.079	0.141
	CD68	0.208	∗∗∗	0.288	∗∗∗
	IL10	0.182	∗∗	0.281	∗∗∗
M1 macrophage	iNOS	0.226	∗∗∗	0.274	∗∗∗
	IRF5	0.362	∗∗∗	0.372	∗∗∗
	COX2	-0.009	0.86	0.089	0.0996
M2 macrophage	CD163	-0.004	0.935	0.077	0.154
	VSIG4	0.035	0.498	0.117	0.0303
	MS4A4A	0.023	0.656	0.125	0.0201
Neutrophils	CD66b	0.102	0.05	0.126	0.0189
	CD11b	0.243	∗∗∗	0.32	∗∗∗
	CCR7	0.036	0.493	0.163	∗
Natural killer cell	KIR2DL1	-0.023	0.665	-0.036	0.504
	KIR2DL3	-0.158	∗	0.2	∗∗
	KIR2DL4	0.216	∗∗∗	0.247	∗∗∗
	KIR3DL1	-0.008	0.88	0.005	0.924
	KIR3DL2	0.095	0.0662	0.129	0.0162
	KIR3DL3	0.076	0.143	0.097	0.0728
	KIR2DS4	0.051	0.332	0.046	0.398
Dendritic cell	HLA-DPB1	0.143	∗	0.25	∗∗∗
	HLA-DQB1	0.147	∗	0.245	∗∗∗
	HLA-DRA	0.131	0.0117	0.236	∗∗∗
	HLA-DPA1	0.095	0.0684	0.203	∗∗
	BDCA-1	0.071	0.175	0.159	∗
	BDCA-4	0.121	0.0199	0.141	∗
	CD11c	0.285	∗∗∗	0.408	∗∗∗
Th1	T-bet	0.069	0.185	0.175	∗
	STAT4	0.21	∗∗∗	0.271	∗∗∗
	STAT1	0.286	∗∗∗	0.335	∗∗∗
	IFN-*γ*	0.278	∗∗∗	0.353	∗∗∗
	TNF-*α*	0.216	∗∗∗	0.34	∗∗∗
Th2	GATA3	0.155	∗	0.274	∗∗∗
	STAT6	0.005	0.926	0.005	0.931
	STAT5A	0.212	∗∗∗	0.254	∗∗∗
	IL13	0.084	0.107	0.094	0.0807
Tfh	BCL6	0.071	0.172	0.075	0.163
	IL21	0.129	0.0132	0.171	∗
Th17	STAT3	-0.023	0.657	0.003	0.963
	IL17A	0.081	0.12	0.1	0.0639
Treg	FOXP3	0.094	0.0713	0.174	∗
	CCR8	0.27	∗∗∗	0.352	∗∗∗
	STAT5B	0.092	0.0763	0.077	0.155
	TGF*β* (TGFB1)	0.259	∗∗∗	0.351	∗∗∗
Exhausted T cells	PD-1 (PDCD-1)	0.347	∗∗∗	0.454	∗∗∗
	CTLA4	0.362	∗∗∗	0.474	∗∗∗
	LAG3	0.364	∗∗∗	0.415	∗∗∗
	TIM3 (HAVCR2)	0.284	∗∗∗	0.427	∗∗∗
	GZMB	0.111	0.0327	0.186	∗∗

HCC: hepatocellular carcinoma; TAM: tumor-associated macrophage; Th1: T-helper 1 cells; Th2: T-helper 2 cells; Th17: T-helper 17 cells; Tfh: follicular helper T cell; Treg: regulatory T cell. Note: partial cor (partial correlation coefficient) represents the correlation coefficient between the CDCA3 expression and gene markers of infiltrating immune cells; purity represents the correlation adjusted by purity; none represents the correlation without adjustment; ^∗^*p* < 0.01, ^∗∗^*p* < 0.001, and ^∗∗∗^*p* < 0.0001.

## Data Availability

The datasets analyzed were acquired from The Cancer Genome Atlas (TCGA) database (https://portal.gdc.cancer.gov/).

## Abbreviations

HCC: Hepatocellular carcinoma
TCGA: The Cancer Genome Atlas
TIMER: Tumor Immune Estimation Resource
TNM: Tumor size/lymph nodes/distance metastasis
CDCA3: Cell division cycle-associated protein-3
GEPIA: Gene Expression Profiling Interactive Analysis
E2Fs: E2F transcription factors
RUNX: Runt-related transcription factor
OS: Overall survival
PFS: Progression-free survival
RFS: Relapse-free survival
DSS: Disease-specific survival
DMFS: Distant metastasis-free survival
PPS: Postprogression survival
FP: First progression
DFS: Disease-free survival
Partial cor: Partial correlation coefficient
TILs: Tumor-infiltrating lymphocytes
TAMs: Tumor-associated macrophages
NK: Natural killer cells
DCs: Dendritic cells
Th1: T-helper 1 cells
Th2: T-helper 2 cells
Tfh: Follicular helper T cells
Th17: T-helper 17 cells
Tome-1: Trigger of mitotic entry
ACC: Adrenocortical carcinoma
BLCA: Bladder urothelial carcinoma
BRCA: Breast invasive carcinoma
CESC: Cervical squamous cell carcinoma and endocervical adenocarcinoma
CHOL: Cholangiocarcinoma
COAD: Colon adenocarcinoma
DLBC: Lymphoid neoplasm diffuse large B cell lymphoma
ESCA: Esophageal carcinoma
GBM: Glioblastoma multiforme
HNSC: Head and neck squamous cell carcinoma
KICH: Kidney chromophobe
KIRC: Kidney renal clear cell carcinoma
KIRP: Kidney renal papillary cell carcinoma
LAML: Acute myeloid leukemia
LGG: Brain lower grade glioma
LIHC (HCC): Liver hepatocellular carcinoma
LUAD: Lung adenocarcinoma
LUSC: Lung squamous cell carcinoma
MESO: Mesothelioma
OV: Ovarian serous cystadenocarcinoma
PAAD: Pancreatic adenocarcinoma
PCPG: Pheochromocytoma and paraganglioma
PRAD: Prostate adenocarcinoma
READ: Rectum adenocarcinoma
SARC: Sarcoma
SKCM: Skin cutaneous melanoma
STAD: Stomach adenocarcinoma
TGCT: Testicular germ cell tumors
THCA: Thyroid carcinoma
THYM: Thymoma
UCEC: Uterine corpus endometrial carcinoma
UCS: Uterine carcinosarcoma
UVM: Uveal melanoma.

## Supplementary Materials

Supplementary 1 Figure S1: correlation between the CDCA3 expression and prognostic curves in diverse types of cancer in the GEPIA databases. Overall survival (OS) and disease-free survival (DFS) curves comparing the high and low expression of CDCA3 in adrenocortical carcinoma (ACC) (a-b), bladder urothelial carcinoma (BLCA) (c-d), breast invasive carcinoma (BRCA) (e-f), cervical squamous cell carcinoma and endocervical adenocarcinoma (CESC) (g-h), cholangiocarcinoma (CHOL) (i-j), colon adenocarcinoma (COAD) (k-l), colon adenocarcinoma (COAD) (m-n), lymphoid neoplasm diffuse large B cell lymphoma (DLBC) (o-p), esophageal carcinoma (ESCA) (q-r), glioblastoma multiforme (GBM) (s-t), head and neck squamous cell carcinoma (HNSC) (u-v), kidney chromophobe (KICH) (w-x), kidney renal clear cell carcinoma (KIRC) (y-z), kidney renal papillary cell carcinoma (KIRP) (aa-ab), acute myeloid leukemia (LAML) (ab-ac), brain lower grade glioma (LGG) (ac-ad), liver hepatocellular carcinoma (LIHC (HCC)), (ae-af), lung adenocarcinoma (LUAD) (ag–ah), lung squamous cell carcinoma (LUSC) (ai–aj), mesothelioma (MESO) (ak–al), ovarian serous cystadenocarcinoma (OV) (am–an), pancreatic adenocarcinoma (PAAD) (ao–ap), pheochromocytoma and paraganglioma (PCPG) (aq–ar), prostate adenocarcinoma (PRAD) (as–at), rectum adenocarcinoma (READ) (au–av), sarcoma (SARC) (aw–ax), skin cutaneous melanoma (SKCM) (ay–az), stomach adenocarcinoma (STAD) (ba–bb), testicular germ cell tumors (TGCT) (bc–bd), thyroid carcinoma (THCA) (be–bf), thymoma (THYM) (bg–bh), uterine corpus endometrial carcinoma (UCEC) (bi–bj), uterine carcinosarcoma (UCS) (bk–bl), and uveal melanoma (UVM) (bm–bn). Note: HR (hazard ratio) represents the ratio of risk rate produced by high CDCA3 expression to the risk rate produced by low CDCA3 expression on survival; logrank *p* < 0.05 was statistically significant; *n*(high) represents the number of patients with high CDCA3 expression; *n*(low) represents the number of patients with low CDCA3 expression; the median of CDCA3 expression was the threshold for splitting highly expressed CDCA3 and lowly expressed CDCA3.

Supplementary 2 Figure S2: correlation of CDCA3 expression with immune infiltration levels in diverse types of cancer in the TIMER database. Adrenocortical carcinoma (ACC) (a), bladder urothelial carcinoma (BLCA) (b), breast invasive carcinoma-basal (BRCA-Basal) (c), breast invasive carcinoma-Her2 (BRCA-Her2) (d), breast invasive carcinoma-luminal (BRCA-Luminal) (e), breast invasive carcinoma (BRCA) (f), cervical squamous cell carcinoma and endocervical adenocarcinoma (CESC) (g), cholangiocarcinoma (CHOL) (h), colon adenocarcinoma (COAD) (i), lymphoid neoplasm diffuse large B cell lymphoma (DLBC) (j), esophageal carcinoma (ESCA) (k), glioblastoma multiforme (GBM) (l), head and neck squamous cell carcinoma-HPVneg (HNSC-HPVneg) (m), head and neck squamous cell carcinoma-HPVpos (HNSC-HPVpos) (n), head and neck squamous cell carcinoma (HNSC) (o), kidney chromophobe (KICH) (p), kidney renal clear cell carcinoma (KIRC) (q), kidney renal papillary cell carcinoma (KIRP) (r), brain lower grade glioma (LGG) (s), lung adenocarcinoma (LUAD) (t), lung squamous cell carcinoma (LUSC) (u), mesothelioma (MESO) (v), ovarian serous cystadenocarcinoma (OV) (w), pancreatic adenocarcinoma (PAAD) (x), pheochromocytoma and paraganglioma (PCPG) (y), prostate adenocarcinoma (PRAD) (z), rectum adenocarcinoma (READ) (aa), sarcoma (SARC) (ab), skin cutaneous melanoma-metastasis (SKCM-Metastasis) (ac), skin cutaneous melanoma-primary (SKCM-Primary) (ad), skin cutaneous melanoma (SKCM) (ae), stomach adenocarcinoma (STAD) (af), testicular germ cell tumors (TGCT) (ag), thyroid carcinoma (THCA) (ah), thymoma (THYM) (ai), uterine corpus endometrial carcinoma (UCEC) (aj), uterine carcinosarcoma (UCS) (ak), and uveal melanoma (UVM) (al). The abundances of six infiltrating immune cells included B cells, CD4+ T cells, CD8+ T cells, neutrophils, macrophages, and dendritic cells. Note: purity represents the tumor purity; partial cor represents the correlation coefficient between infiltrating immune cells and the expression of CDCA3; the higher the value of partial cor, the stronger the correlation; *p* < 0.05 was statistically significant.

Supplementary 3 Table S1: CDCA3 expression in cancers versus normal tissue in the ONCOMINE database. Note: the results were shown by rank (%), *p* value, fold changes, and the number of samples including tumor tissue samples and normal tissue samples; the teratoma-NOS represents the nonspecific type of teratoma.
