# Supplementary figures and images for: CDCA3 Is a Novel Prognostic Biomarker Associated with Immune Infiltration in Hepatocellular Carcinoma
Source: Biomed Res Int. 2021 Jan 29;2021:6622437. doi: 10.1155/2021/6622437 (PMC7869413; doi:10.1155/2021/6622437)

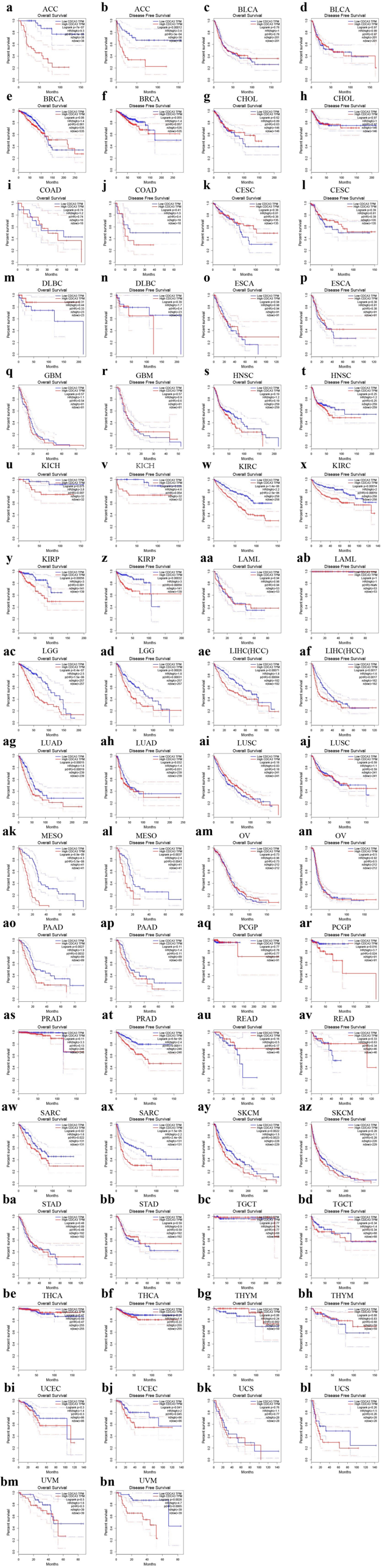

Supplement: Supplementary 1 — Figure S1: correlation between the CDCA3 expression and prognostic curves in diverse types of cancer in the GEPIA databases. Overall survival (OS) and disease-free survival (DFS) curves comparing the high and low expression of CDCA3 in adrenocortical carcinoma (ACC) (a-b), bladder urothelial carcinoma (BLCA) (c-d), breast invasive carcinoma (BRCA) (e-f), cervical squamous cell carcinoma and endocervical adenocarcinoma (CESC) (g-h), cholangiocarcinoma (CHOL) (i-j), colon adenocarcinoma (COAD) (k-l), colon adenocarcinoma (COAD) (m-n), lymphoid neoplasm diffuse large B cell lymphoma (DLBC) (o-p), esophageal carcinoma (ESCA) (q-r), glioblastoma multiforme (GBM) (s-t), head and neck squamous cell carcinoma (HNSC) (u-v), kidney chromophobe (KICH) (w-x), kidney renal clear cell carcinoma (KIRC) (y-z), kidney renal papillary cell carcinoma (KIRP) (aa-ab), acute myeloid leukemia (LAML) (ab-ac), brain lower grade glioma (LGG) (ac-ad), liver hepatocellular carcinoma (LIHC (HCC)), (ae-af), lung adenocarcinoma (LUAD) (ag–ah), lung squamous cell carcinoma (LUSC) (ai–aj), mesothelioma (MESO) (ak–al), ovarian serous cystadenocarcinoma (OV) (am–an), pancreatic adenocarcinoma (PAAD) (ao–ap), pheochromocytoma and paraganglioma (PCPG) (aq–ar), prostate adenocarcinoma (PRAD) (as–at), rectum adenocarcinoma (READ) (au–av), sarcoma (SARC) (aw–ax), skin cutaneous melanoma (SKCM) (ay–az), stomach adenocarcinoma (STAD) (ba–bb), testicular germ cell tumors (TGCT) (bc–bd), thyroid carcinoma (THCA) (be–bf), thymoma (THYM) (bg–bh), uterine corpus endometrial carcinoma (UCEC) (bi–bj), uterine carcinosarcoma (UCS) (bk–bl), and uveal melanoma (UVM) (bm–bn). Note: HR (hazard ratio) represents the ratio of risk rate produced by high CDCA3 expression to the risk rate produced by low CDCA3 expression on survival; logrank p < 0.05 was statistically significant; n(high) represents the number of patients with high CDCA3 expression; n(low) represents the number of patients with low CDCA3 expression; the med [file 6622437.f1.pdf]

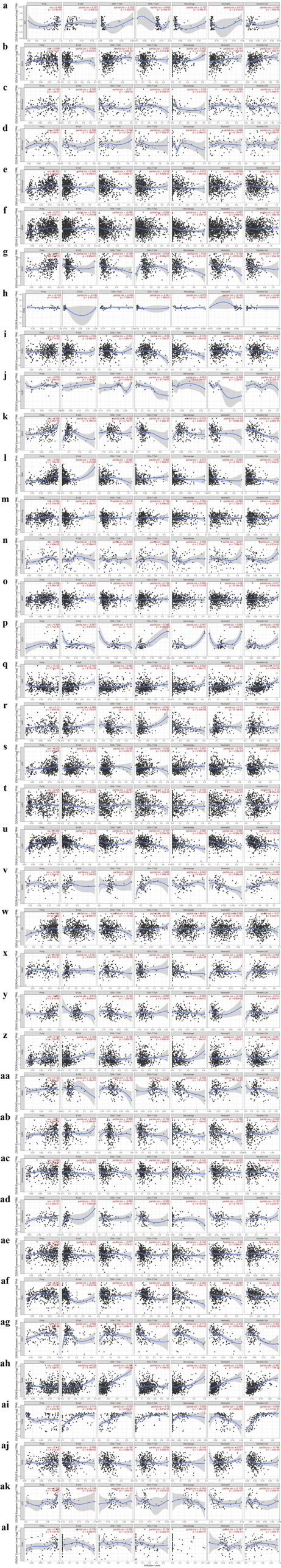

Supplement: Supplementary 2 — Figure S2: correlation of CDCA3 expression with immune infiltration levels in diverse types of cancer in the TIMER database. Adrenocortical carcinoma (ACC) (a), bladder urothelial carcinoma (BLCA) (b), breast invasive carcinoma-basal (BRCA-Basal) (c), breast invasive carcinoma-Her2 (BRCA-Her2) (d), breast invasive carcinoma-luminal (BRCA-Luminal) (e), breast invasive carcinoma (BRCA) (f), cervical squamous cell carcinoma and endocervical adenocarcinoma (CESC) (g), cholangiocarcinoma (CHOL) (h), colon adenocarcinoma (COAD) (i), lymphoid neoplasm diffuse large B cell lymphoma (DLBC) (j), esophageal carcinoma (ESCA) (k), glioblastoma multiforme (GBM) (l), head and neck squamous cell carcinoma-HPVneg (HNSC-HPVneg) (m), head and neck squamous cell carcinoma-HPVpos (HNSC-HPVpos) (n), head and neck squamous cell carcinoma (HNSC) (o), kidney chromophobe (KICH) (p), kidney renal clear cell carcinoma (KIRC) (q), kidney renal papillary cell carcinoma (KIRP) (r), brain lower grade glioma (LGG) (s), lung adenocarcinoma (LUAD) (t), lung squamous cell carcinoma (LUSC) (u), mesothelioma (MESO) (v), ovarian serous cystadenocarcinoma (OV) (w), pancreatic adenocarcinoma (PAAD) (x), pheochromocytoma and paraganglioma (PCPG) (y), prostate adenocarcinoma (PRAD) (z), rectum adenocarcinoma (READ) (aa), sarcoma (SARC) (ab), skin cutaneous melanoma-metastasis (SKCM-Metastasis) (ac), skin cutaneous melanoma-primary (SKCM-Primary) (ad), skin cutaneous melanoma (SKCM) (ae), stomach adenocarcinoma (STAD) (af), testicular germ cell tumors (TGCT) (ag), thyroid carcinoma (THCA) (ah), thymoma (THYM) (ai), uterine corpus endometrial carcinoma (UCEC) (aj), uterine carcinosarcoma (UCS) (ak), and uveal melanoma (UVM) (al). The abundances of six infiltrating immune cells included B cells, CD4+ T cells, CD8+ T cells, neutrophils, macrophages, and dendritic cells. Note: purity represents the tumor purity; partial cor represents the correlation coefficient between infiltrating immune cells and the expressio [file 6622437.f2.pdf]
