# Supplementary material for: CDCA3 Is a Novel Prognostic Biomarker Associated with Immune Infiltration in Hepatocellular Carcinoma
Source: Biomed Res Int. 2021 Jan 29;2021:6622437. doi: 10.1155/2021/6622437 (PMC7869413; doi:10.1155/2021/6622437)
Supplement: Supplementary 3 — Table S1: CDCA3 expression in cancers versus normal tissue in the ONCOMINE database. Note: the results were shown by rank (%), p value, fold changes, and the number of samples including tumor tissue samples and normal tissue samples; the teratoma-NOS represents the nonspecific type of teratoma. [file 6622437.f3.docx]

**Table S1. CDCA3 expression in cancers verus normal tissue in ONCOMINE database.**

| **Cancer** | Cancer type | *p* value | Fold change | Rank (%) | Sample | Normal sample | Tumor sample | Reference (PMID) |
| --- | --- | --- | --- | --- | --- | --- | --- | --- |
| **Bladder** | Infiltrating Bladder Urothelial Carcinoma | 2.29E-12 | 2.625 | 1% | 130 | 68 | 62 | 20421545 |
|  | Superficial Bladder Cancer | 1.43E-11 | 5.595 | 9% | 76 | **48** | **28** | **16432078** |
| **Brain and CNS** | Glioblastoma | 8.68E-21 | 5.670 | 1% | 104 | 23 | 81 | 16616334 |
| **Breast** | Medullary Breast Carcinoma | 2.19e-14 | 3.854 | 1% | 176 | 144 | 32 | 22522925 |
|  | Ductal Breast Carcinoma | 4.53E-12 | 9.115 | 1% | 47 | 7 | 40 | 16473279 |
|  | Ductal Breast Carcinoma in Situ Epithelia | 4.35E-5 | 2.127 | 2% | 23 | 14 | 9 | 19187537 |
|  | Lobular Breast Carcinoma | 8.37E-6 | 2.174 | 3% | 23 | 3 | 20 | 15034139 |
|  | Invasive Breast Carcinoma Stroma | 1.77E-16 | -5.256 | 10% | 59 | 6 | 53 | 18438415 |
| **Cervical** | Cervical Squamous Cell Carcinoma | 1.34E-7 | 3.465 | 3% | 56 | 24 | 32 | 18523997 |
|  | Cervical Cancer | 9.71E-7 | 2.153 | 9% | 42 | **22** | **20** | **17510386** |
| **Colorectal** | Rectosigmoid Adenocarcinoma | 2.82E-10 | 2.635 | 1% | 25 | 22 | 3 | TCGA |
|  | Rectal Mucinous Adenocarcinoma | 6.53E-6 | 2.304 | 3% | 28 | 22 | 6 | TCGA |
|  | Cecum Adenocarcinoma | 8.24E-12 | 2.025 | 3% | 44 | 22 | 22 | TCGA |
|  | Rectal Adenocarcinoma | 1.87E-12 | 2.036 | 8% | 82 | 22 | 60 | TCGA |
|  | Colon Adenoma | 6.65E-17 | 2.856 | 1% | 57 | **32** | **25** | **18171984** |
|  | Rectal Adenoma | 8.99E-7 | 4.512 | 3% | 39 | **32** | **7** | **18171984** |
|  | Rectal Adenocarcinoma | 6.18E-32 | 3.245 | 1% | 130 | **65** | **65** | **20725992** |
|  | Colon Carcinoma | 3.69E-9 | 2.882 | 3% | 15 | **10** | **5** | **20957034** |
|  | Colon Adenoma | 1.25E-5 | 2.518 | 6% | 15 | 10 | 5 | 20957034 |
|  | Colorectal Carcinoma | 7.68E-9 | 3.677 | 7% | 82 | **12** | **70** | **20143136** |
| **Esophageal** | Esophageal Squamous Cell Carcinoma | 9.72E-7 | 2.188 | 4% | 34 | 17 | 17 | 20955586 |
| **Gastric** | Diffuse Gastric Adenocarcinoma | 2.19E-5 | 2.106 | 4% | 50 | 19 | 31 | 21447720 |
|  | Gastric Intestinal Type Adenocarcinoma | 5.23E-9 | 3.275 | 5% | 57 | **31** | **26** | **19081245** |
| Leukemia | Acute Myeloid Leukemia | 2.10E-7 | -2.949 | 5% | 29 | 6 | 23 | 17544290 |
| Liver | Hepatocellular Carcinoma | 3.39E-8 | 3.241 | 2% | 45 | 10 | 35 | 17393520 |
| **Lung** | Lung Adenocarcinoma | 2.60E-11 | 4.143 | 1% | 57 | 30 | 27 | 17540040 |
|  | Squamous Cell Lung Carcinoma | 5.79E-26 | 7.717 | 1% | 92 | **65** | **27** | **20421987** |
|  | Lung Adenocarcinoma | 9.34E-16 | 3.551 | 1% | 110 | **65** | **45** | **20421987** |
|  | Large Cell Lung Carcinoma | 1.08E-8 | 4.431 | 1% | 84 | **65** | **19** | **20421987** |
| Lymphoma | Unspecified Peripheral T-Cell Lymphoma | 1.32E-10 | 3.853 | 6% | 48 | **20** | **28** | **17304354** |
|  | Angioimmunoblastic T-Cell Lymphoma | 3.04E-5 | 2.858 | 8% | 26 | **20** | **6** | **17304354** |
| Melanoma | Cutaneous Melanoma | 4.24E-15 | 8.783 | 1% | 52 | **7** | **45** | **16243793** |
|  | Benign Melanocytic Skin Nevus | 2.12E-5 | 2.439 | 3% | 25 | **7** | **18** | **16243793** |
| Sarcoma | Fibrosarcoma | 4.77E-7 | 6.005 | 1% | 22 | **15** | **7** | **15994966** |
|  | Pleomorphic Liposarcoma | 5.73E-6 | 4.707 | 1% | 18 | **15** | **3** | **15994966** |
|  | Malignant Fibrous Histiocytoma | 6.04E-7 | 5.825 | 1% | 24 | **15** | **9** | **15994966** |
|  | Leiomyosarcoma | 1.33E-5 | 5.333 | 2% | 21 | **15** | **6** | **15994966** |
| **Others** | Malignant Fibrous Histiocytoma | 6.04E-7 | 5.825 | 1% | 24 | **15** | **9** | **15994966** |
|  | Testicular Seminoma | 2.40E-7 | 2.705 | 4% | 38 | **19** | **19** | **14595015** |
|  | Skin Squamous Cell Carcinoma | 2.90E-5 | 2.593 | 3% | 15 | **4** | **11** | **18442402** |
|  | Vulvar Intraepithelial Neoplasia | 2.08E-5 | 3.840 | 3% | 19 | **10** | **9** | **17471573** |
|  | Teratoma, NOS | 3.40E-7 | -2.533 | 10% | 20 | **6** | **14** | **16424014** |

Note: Teratoma, NOS represents the nonspecific type of Teratoma; the results were showed by rank (%), *p* value, fold changes and the number of samples including tumor tissues samples and normal tissues samples.
